# Cystoid macular oedema in Cogans syndrome-a case report

**DOI:** 10.1186/1757-1626-1-339

**Published:** 2008-11-20

**Authors:** Anupama Pherwani, Shveta Bansal, Shailesh Agrawal, Timothy Gillow

**Affiliations:** 1 North Staffordshire University Hospital, Stoke On Trent, UK; 2 St James' University Hospital, Leeds, UK; 3 Hope Hospital, Manchester, UK

## Abstract

**Introduction:**

Cogan's Syndrome is typically characterised by a non syphilitic interstitial keratitis (IK), with or without conjunctivitis, iritis or subconjunctival bleeding and progressive sensorineural hearing loss within two years of ocular signs. Atypical ocular manifestations include episcleritis, scleritis, posterior scleritis, retinal artery occlusion, choroiditis, retinal vasculitis, and optic disc oedema. We report a case of Cogan's syndrome in with recurrent cystoid macular oedema was the main feature.

**Case presentation:**

A patient was diagnosed with Cogan's syndrome nearly 2 years after first presentation. He had cystoid macular edema which failed to respond initially to steroid, methotrexate and azothiaprine however resolved after treatment with mycophenolate mofetil.

**Conclusion:**

Cogan's syndrome is rare and presents a challenge in terms of diagnosis and treatment. Recurrent cystoid macular oedema is a unique finding in this condition and can be difficult to control. Multidisciplinary management of this multisystem disorder is vital.

## Introduction

David Cogan first described typical Cogan's syndrome in 1945. It presents with non syphilitic interstitial keratitis (IK), with or without conjunctivitis, iritis or subconjunctival bleeding and Menier's like audiovestibular signs associated with progressive sensorineural hearing loss within two years of ocular signs [1] Atypical Cogan's encompasses other ocular or audiovestibular symptoms or a delay of more than 2 years between them [2]. Atypical ocular manifestations include episcleritis, scleritis, posterior scleritis, retinal artery occlusion, choroiditis, retinal vasculitis, and optic disc oedema with or without IK [3,4]

Cystoid macular edema (CMO) in Cogan's syndrome has not been described before. Vollertsen et al in their review report macular involvement, but do not describe the pathology seen at the macula [5]. We report a case of Cogan's syndrome in which recalcitrant CMO was the main feature.

## Case presentation

A 54 year old Caucasian man presented to eye casualty with a history of blurred vision and grittiness in his right eye (RE) for over six months. Snellen visual acuity was 6/5 in both eyes. Examination revealed mild injection and a stromal opacity with deep vascularisation in the superior aspect of the right cornea. There was no associated inflammation in the anterior chamber and corneal sensation was normal. Examination of the left eye was unremarkable. A diagnosis of probable non necrotising herpetic stromal keratitis was made and the eye treated with topical prednisolone acetate 0.5%. Three weeks later, the patient was asymptomatic and the injection had resolved. The topical treatment was tapered and discontinued.

The patient, re-attended 3 months later with similar symptoms along with acute onset tinnitus in the right ear and deafness of 3 weeks duration. Ocular examination revealed the corneal stromal opacity, which was now diagnosed as interstitial keratitis. There were no signs of active inflammation in the eye. Blood tests were performed and a high ESR (88 mm at 1 hour) was the only positive finding. All autoantibodies including ANA, cANCA, pANCA were negative, serum ACE was within normal limits, no antibodies were found against treponema pallidum and borrelia bergdorfi. The patient was referred to an otorhinologist, who performed an audiogram, which revealed bilateral sensorineural deafness.

**Figure 1:**
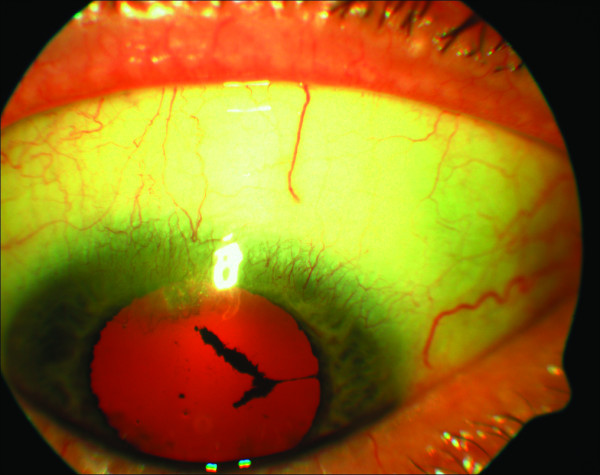
Right cornea showing stromal opacity with vascularisation in the superior aspect.

**Figure 2:**
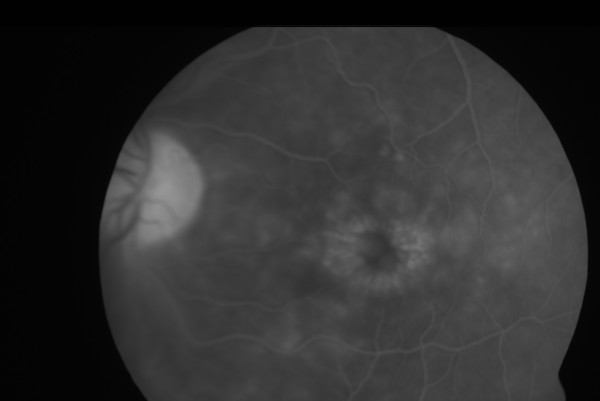
Figures 2 and 3: Fundus flourescein angiogram showing classical appearance of cystoid macular oedema in the left eye (fig 2) and right eye (fig 3)

**Figure 3:**
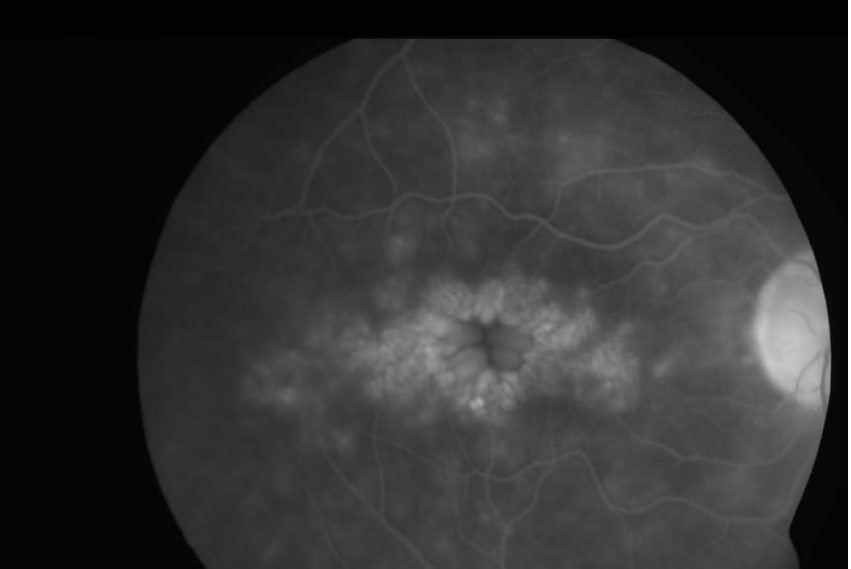
Figures 2 and 3: Fundus flourescein angiogram showing classical appearance of cystoid macular oedema in the left eye (fig 2) and right eye (fig 3)

Two years later the patient presented with blurred and distorted vision in both eyes. Aided Snellen's acuity was 6/9 bilaterally. The right cornea still had the deep stromal opacity with ghost vessels superiorly (fig 1). There was mild anterior uveitis. There was mild anterior uveitis and bilateral cystoid macular edema (CMO) (fig 2 and 3). Topical steroids and ketorolac were commenced. Blood tests revealed a raised ESR, serology for autoantibodies, Treponema and Borrelia, serum ACE, chest X ray were normal.

A diagnosis of Cogan's syndrome was made after taking into account the history, clinical findings and results of investigations. The visual acuity over the next 2 weeks deteriorated to 6/18 in the RE and 6/12 in the LE. Oral Prednisolone (40 mg/day) was commenced as treatment. The CMO resolved and hearing improved 2 weeks following treatment. However, once Prednisolone was tapered to 10 mg per day, the CMO recurred. Over a period of 1 year, the CMO fluctuated in severity inversely to the dose of steroids. Triamcinolone acetonide (40 mg) was injected into the orbital floor on two occasions with no benefit. The patient developed secondary posterior subcapsular cataract in the RE and steroid induced diabetes mellitus. He was therefore commenced on intramuscular methotrexate. The CMO did not respond to methotrexate, this was therefore substituted with Azathioprine 125 mg daily. The patient was intolerant to the azathioprine and was commenced on Mycophenolate mofetil. CMO finally settled with Mycophenolate mofetil which enabled tapering off the oral steroid. There was no further deterioration of vision and no recurrence of CMO over 6 months with snellen visual acuity stabilising at 6/24 in the RE (6/12 with pinhole) and 6/9 in the LE.

## Discussion

Cogan's syndrome is a rare auto immune disorder with ocular, auditory and systemic manifestations. It appears to occur in young adult Caucasians of either sex. The ocular involvement is variable, usually bilateral and can cause moderate to severe visual loss. Vollertsen et al reported blindness in 8 out of 156 eyes, with bilateral loss of vision in 2 patients [5].

The disease course of Cogan's syndrome may vary with some patients experiencing an initial flare lasting weeks or months followed by slow progression, whilst others display recurrent ocular or vestibular flare ups. Permanent deafness and death due to systemic manifestations may also occur.

The aetiology and pathogenesis of Cogan's syndrome is unclear. An autoimmune basis has been suggested with description of autoantibodies against the inner ear and endothelium [6]. Diagnosis however is based on clinical findings and history.

Corticosteroids are the first line of treatment and it is argued that if given early in the disease course can aid recovery of the hearing. Immunosupressive drugs like methotrexate, azathioprine, cyclosporine and cyclophosphamide have all been tried with varying degree of success [7-10]. However in the absence of controlled trials, there are no definitive therapeutic recommendations.

## Conclusion

This case highlights the variability of presentation of this rare syndrome and to our knowledge is the first described case of Cogan's syndrome with recurring cystoid macular oedema. Delay in diagnosis is common and can lead to visual and auditory disability. It is a multisystem disease, which can be sight threatening and aggressive therapeutic intervention may be needed to control the ocular pathology and prevent visual loss. Optimum management of these patients needs close co-operation of the ophthalmologist, otorhinolaryngologist and physician.

## Consent

The authors obtained written informed consent from the parents of this patient for the publication of this case report along with images. A copy of the written consent is available for review by the Editor-in-Chief of this journal.

## Competing interests

The authors declare that they have no competing interests.

## Authors' contributions

AP was the lead author contributing to carrying out the literature search, study design and writing the case report. SB and SA assisted in the writing of the paper, study design and literature search. SA also helped in interpretation of audiogram results. TG supervised the management of the case, assisted in study design and writing of the paper. All authors have been involved in approving the final manuscript.
